# Review: Advances in the Pathogenesis and Treatment of Immune Thrombocytopenia Associated with Viral Hepatitis

**DOI:** 10.1055/s-0043-1772771

**Published:** 2023-08-24

**Authors:** Yanmei Xu, Yunfei Chen, Lei Zhang

**Affiliations:** 1 State Key Laboratory of Experimental Hematology, National Clinical Research Center for Blood Diseases, Haihe Laboratory of Cell Ecosystem, Institute of Hematology & Blood Diseases Hospital, Chinese Academy of Medical Sciences & Peking Union Medical College, Tianjin Key Laboratory of Gene Therapy for Blood Diseases, CAMS Key Laboratory of Gene Therapy for Blood Diseases, Tianjin, People's Republic of China; 2 Tianjin Institutes of Health Science, Chinese Academy of Medical Science & Peking Union Medical College, Tianjin, People's Republic of China

**Keywords:** viral hepatitis, immune thrombocytopenia, pathogenesis, treatment

## Abstract

Hepatitis B virus and hepatitis C virus are the hepatitis subtypes that most commonly induce immune thrombocytopenia (ITP). Although the pathogenesis of viral hepatitis-associated ITP remains unclear, it may involve antibody cross-reactivity due to molecular mimicry, the formation of virus-platelet immune complexes, and T cell-mediated suppression of bone marrow hematopoiesis. Moreover, there is significant correlation between platelet count and the severity of viral hepatitis, the risk of progression to liver cirrhosis, and clinical prognosis. However, treatment of viral hepatitis-associated ITP is hindered by some antiviral drugs. In this review, we summarize research progress to date on the pathogenesis and treatment of viral hepatitis-related ITP, hoping to provide a reference for clinical diagnosis and treatment.

## Introduction

Immune thrombocytopenia (ITP) is a kind of acquired autoimmune disease mainly caused by autoantibody-mediated excessive destruction of platelets by the reticuloendothelial system and impaired platelet maturation and production by megakaryocytes due to immune intolerance in patients. ITP can be induced by a variety of clinical conditions, such as viral infection (human immunodeficiency virus [HIV], cytomegalovirus, hepatitis virus), systemic lupus erythematosus, lymphoproliferative disease, and certain drugs or vaccinations. Although hepatitis A virus has been reported to induce thrombocytopenia, the most common clinical cases are ITP associated with hepatitis B or C. According to one study, the incidence of ITP associated with hepatitis C virus (HCV) and hepatitis B virus (HBV) infection is 11.86 and 6.35%, respectively. 1 Approximately 30% of adults with chronic ITP have anti-HCV antibodies in their serum. 2 In addition, the initial symptoms of HBV or HCV infection are insidious and may only manifest as a decrease in peripheral blood platelet count, which poses a great challenge for clinical diagnosis and treatment. Here, we summarize the results of studies on the pathogenesis and treatment of hepatitis-related ITP and to provide a theoretical reference for clinical diagnosis and treatment.

## Epidemiology

Hepatitis virus infection is distributed differently worldwide, with the most common in China being infected with HBV and/or HCV. Epidemiological data for China show a prevalence of hepatitis B surface antigen (HBsAg) in the general population of 5 to 6% and ∼70 million cases of chronic HBV infection, including 20 to 30 million cases of chronic HBV infection. 3 Furthermore, the results of a survey in 2014 revealed the number of HCV infections in China to be as high as 29.8 million, ranking first in the world. 4

Hepatitis viruses are pantropic viruses that can affect the hematopoietic system at different stages of the disease course. Although the incidence of HCV infection is much lower than that of HBV, the former is more likely to be accompanied by ITP during the course of the disease. 5 Indeed, the incidence of thrombocytopenia in HCV-infected patients is 30.2 per 100,000, nearly twice as high as that in the HCV antibody-negative population. In patients with ITP, the presence of HCV increases the degree of thrombocytopenia and exacerbates bleeding tendencies. 2 6 The results of our local study showed that 7.3% of patients diagnosed with ITP had HBV or HCV infection, with 65% of infections being detected for the first time. 7 This not only suggests a close relationship between viral hepatitis and ITP but also reflects the insidious nature of the course of viral hepatitis.

## Pathogenesis of Viral Hepatitis-Associated ITP

Thrombocytopenia is the most common extrahepatic manifestation of viral hepatitis and is usually caused by a combination of several factors. Although the exact pathogenesis of ITP associated with viral hepatitis has not been elucidated, the mechanisms suggested by studies to date are described later.

### Molecular Simulation Hypothesis

The molecular mimicry hypothesis is one of the oldest theories of autoimmune initiation. This theory proposes that a molecule in the environment that has structural similarity to a host's molecule can induce the host to produce antibodies that may cross-react with the host's antigens and thus cause an autoimmune response. 8 It has been suggested that HCV infection may induce cross-reactivity between immunoglobulin G (IgG) antibodies and the platelet membrane glycoprotein (GP)IIIa and that binding of IgG to GPIIIa activates NADPH oxidase in platelets, causing them to produce large amounts of reactive oxygen species, which promotes their apoptosis. 9 10 Such cross-reactivity of antibodies caused by HCV is not unique, as HIV and quinine also induce platelet reduction in similar ways. 11 Furthermore, several studies have reported that the presence of platelet-associated antibodies (PAIgG, PAIgM) can be detected in patients with chronic HBV or HCV infection and thrombocytopenia; the detection rate of platelet-associated antibodies in HCV-infected patients is 80%, much higher than in HBV-infected patients. 12 13 Overall, HBV/HCV infection activates immune cells to eliminate the virus, and many products (antibodies, inflammatory cytokines, chemokines) released during this process might lead to immune dysfunction and even induce autoimmune disease.

### Increased Platelet Clearance Mediated by Virus–Platelet Immune Complexes

In addition to the hemostatic effect, platelets possess immunomodulatory functions. Studies have shown that HCV can directly bind to the collagen receptor GPVI or to dendritic cell-specific intercellular adhesion molecule-3-grabbing nonintegrin, which is expressed on the platelet surface, leading to platelet activation and release of a variety of inflammatory cytokines and chemokines that mediate viral clearance and platelet exhaustion. 14 15 Moreover, platelets express a low-affinity receptor for the constant domain of IgG-FcγIIA on their surface, through which virus-containing immune complexes bind and accelerate platelet clearance.

Interestingly, there is a parasitic relationship between hepatitis viruses and platelets. de Almeida et al 16 reported a significantly high rate of HCV RNA detection in platelet samples from patients with thrombocytopenia, suggesting that platelets can act as a reservoir for HCV to help it evade immune clearance. After HBV infects hepatocytes, the exposed tissue factor and von Willebrand factor activate platelets and recruit neutrophils, promoting the formation of an inflammatory microenvironment in liver sinusoids. In the inflammatory environment, Kupffer cells in hepatic sinusoids continuously phagocytose platelet-neutrophil aggregates, causing a dramatic decrease in the circulating platelet count. 17 In short, the complex interaction between platelets and hepatitis viruses makes it inevitable that the monocyte-macrophage system will lead to accelerated destruction of platelets when it attacks hepatitis viruses, which probably affects the peripheral blood platelet count and induces bleeding.

### Suppression of Bone Marrow Hematopoiesis by Abnormally Activated T Cells

An in vitro study published by Zeldis et al 18 in 1986 showed that the colony-forming capacity of bone marrow hematopoietic stem cells (HSCs) cultured in HBV-containing serum was reduced and that removal of HBV by inactivation or adsorption corrected this defect. This study demonstrated that in addition to hepatocytes, hepatitis viruses can directly affect the function of bone marrow HSCs. Since then, HBV gene fragments and their protein products have been identified in HSCs, confirming that hepatitis viruses can infect HSCs, with integration of their genetic material into the HSC genome. HBsAg secreted by infected HSCs acts as an endogenous antigen to activate T cell-mediated HSC damage and inhibit HSC proliferation and differentiation. 19 20 21 Although the exact mechanism by which hepatitis viruses directly damage HSCs remains to be elucidated, we found the immune response and cellular damage induced by hepatitis virus infection to be extensive. The occurrence of hepatitis virus-associated ITP involves the combination of a reduction in platelet production and an increase in platelet destruction, with a mechanism including multiple links between humoral and cellular immunities.

## Treatment

HBV and HCV infection can lead to severe platelet hypoplasia before progression to the cirrhotic stage, and these patients are more prone to bleeding than those with primary ITP. Therefore, it is important to focus on treatment of thrombocytopenia along with the reduction in viral load through antiviral and other drugs. There is no international consensus on the selection of drugs for treatment of hepatitis-related ITP; in clinical practice, drugs recommended by guidelines related to primary ITP to enhance platelet levels are mostly considered.

### Glucocorticoids and Human Immunoglobulin

Glucocorticoids and human immunoglobulin are the first-line treatment for primary ITP, with initial response rates reach 80%. Based on data from several clinical studies, the response rate to steroids in patients with hepatitis-associated ITP is 20 to 50%, which is much lower than that of patients with primary ITP. 2 22 23 Moreover, long-term use of steroids tends to increase hepatitis virus replication and may aggravate liver damage. 24 Treatment of HCV-infected patients with steroids combined with interferon (IFN)-α is effective at reducing the viral load, and an increase in platelet count after treatment can occur in some cases. 25 However, IFN-α itself can induce ITP, and this regimen is often suggested for clinical treatment of those with chronic severe hepatitis. 26 27 Intravenous immunoglobulin (IVIG) can significantly elevate platelet counts in patients with hepatitis-associated ITP and is generally only recommended for short-term use to reduce bleeding.

### Platelet-Promoting Drugs

Recombinant human thrombopoietin (rhTPO) and thrombopoietin receptor agonists (TPO-RAs) can promote the proliferation and differentiation of bone marrow HSCs by exogenously supplementing or mimicking TPO action with TPO receptors expressed on the surface of these cells. The combination of TPO and TPO receptor activates the downstream JAK-STAT signaling pathway to replenish platelets at the source. Comparison of platelet counts and bleeding symptoms in patients with hepatitis B-related cirrhosis before and after administration of prednisone, rhTPO, or prednisone + rhTPO or with no treatment of rhTPO was shown to effectively elevate platelet levels, and this effect was markedly enhanced when combined with prednisone. 28

The TPO receptor agonists currently used in clinical practice mainly include eltrombopag, romiplostim, and avatrombopag. McHutchison et al 29 first reported the results of a randomized controlled study of eltrombopag for patients with HCV-associated cirrhosis in 2007. After 4 weeks of treatment, patients in the eltrombopag group experienced dose-dependent increases in platelet count, with a significantly higher proportion of patients who completed 12 weeks of antiviral therapy, compared with the placebo group (36–65 vs. 6%). Subsequent findings indicated that TPO-RAs are effective at elevating platelet counts in patients with viral hepatitis-related cirrhosis, reducing the number of platelet transfusions before and after invasive treatment or surgery and decreasing the risk of postoperative bleeding. In addition, TPO-RAs contribute to the initiation and maintenance of antiviral drugs (IFN, ribavirin, etc.), enabling more patients to achieve a sustained virological response. 30 31 32

### Splenic Artery Embolization or Splenectomy

The spleen is the site of platelet destruction and clearance, and platelet counts can be significantly elevated by splenic artery embolization or splenectomy. This measure is often recommended for patients with viral hepatitis-associated thrombocytopenia who failed to respond to steroids, TPO-RAs, or IVIG, particularly among those with hypersplenism. The results from a study conducted by Akahoshi et al 33 showed that mean platelet counts at 1 month after splenectomy in patients with HCV-associated thrombocytopenia increased by more than 200% over baseline. The function of partial splenic artery embolization is similar to that of splenectomy, though it is associated with a lower risk of postoperative infection and portal vein thrombosis and may be an alternative to splenectomy. 34

### Immunosuppressants

A Chinese study group used cyclosporine in combination with nucleoside analogs to enhance platelet levels in patients with HBV-related cirrhosis and achieved good results. Although the regimen did not increase the rate of HBV-DNA conversion, platelet counts were higher in the treated patients than in the control group, and the risk of bleeding was effectively controlled. 35 It should be noted that immunosuppressive therapy can impair the immune response of patients; hepatitis viruses that have not been cleared can replicate and infect hepatocytes in large numbers, which is termed hepatitis virus “reactivation.” 36 37 Clinically, immunosuppressive therapy for viral hepatitis-related thrombocytopenia is rarely employed but may be considered for treating refractory cases with severe bleeding.

## Summary

Overall, the causes of ITP induced by viral hepatitis remain unclear. The possible mechanisms include three aspects: (1) antibody cross-reactivity due to molecular mimicry, (2) the formation of virus–platelet immune complexes, and (3) the T cell-mediated suppression of bone marrow hematopoiesis. Platelet counts in patients with chronic viral hepatitis are critically related to the degree of liver fibrosis and the effect of drug therapy. 38 39 In addition, patients with hepatitis usually have coagulation disorders, which, combined with elevated portal pressure and thrombocytopenia, eventually result in a significantly higher risk and extent of bleeding than in other diseases associated with thrombocytopenia. Therefore, it is essential to elevate the platelet count of patients while administering antiviral therapy.

Although several studies have proposed that antiviral drugs increase platelet counts in patients with HBV or HCV infection, these drugs are often contraindicated in those with severe thrombocytopenia (<50 × 10 ^9^ /L). 40 Currently, treatments recommended for viral hepatitis associated with ITP include glucocorticoids, rhTPO, TPO-RAs, and splenectomy. The efficacy of TPO-RAs in hepatitis-associated thrombocytopenia has been widely recognized in clinical trial studies. Furthermore, the availability of TPO-RAs has increased the proportion of patients who can complete the entire course of antiviral therapy and reduce the incidence of hepatitis-associated cirrhosis. It should be emphasized that the potent ability of TPO-RAs to elevate platelet levels also increases the risk of thrombosis and that adequate thrombotic risk assessment should be performed before drug administration. In addition to the risk of thrombosis, the most concerning problem of TPO-RAs for treatment of primary ITP is the platelet rebound phenomenon after drug withdrawal. Of course, the most important cause of viral hepatitis-related ITP is viral infection. Combined antiviral drugs can reduce or even eliminate viral load in patients as much as possible, and the symptoms of thrombocytopenia will be significantly improved. When predisposing factors are removed, TPO-RAs may achieve better efficacy in viral hepatitis-related ITP than in primary ITP. Nevertheless, studies on dose reduction, maintenance, and even discontinuation of TPO-RAs in treating those with refractory and severe hepatitis-associated thrombocytopenia are necessary.

Splenectomy is considered the only potentially curative treatment for ITP. Especially for patients with chronic viral hepatitis, decompensated cirrhosis usually occurs in the later stage of disease development, readily leading to complications such as hypersplenism and portal hypertension. Surgical resection of an enlarged spleen will not only reduce retention of platelets in the spleen but also portal vein pressure and improve systemic symptoms. However, removal of the spleen can increase the risk of infection and thrombosis, and patients with viral hepatitis have impaired immune function and coagulation function disorder, which increases the risk of complications after splenectomy. Therefore, indications for splenectomy in viral hepatitis-related ITP need to be judged by clinicians under prudent consideration of disease status and the patient's physical conditions.

In conclusion, the mechanisms involved in viral hepatitis-associated ITP are complex, and diagnosis should be clarified clinically with the aid of antiplatelet antibodies, thrombopoietin, and viral serology tests. Therapeutic drugs should be used to achieve the best clinical benefit for patients based on a dual approach of antivirus and increasing platelet counts as much as possible.
